# Novel subharmonic-aided pressure estimation for identifying high-risk esophagogastric varices

**DOI:** 10.1007/s00535-024-02161-4

**Published:** 2024-10-29

**Authors:** Hidekatsu Kuroda, Tamami Abe, Naohisa Kamiyama, Takuma Oguri, Asami Ito, Ippeki Nakaya, Takuya Watanabe, Hiroaki Abe, Kenji Yusa, Yudai Fujiwara, Hiroki Sato, Akiko Suzuki, Kei Endo, Yuichi Yoshida, Takayoshi Oikawa, Keisuke Kakisaka, Kei Sawara, Akio Miyasaka, Takayuki Matsumoto

**Affiliations:** 1 Division of Gastroenterology and Hepatology, Department of Internal Medicine, Iwate Medical University, Iwate Medical University School of Medicine, Nishitokuta 2-1-1, Yahaba-Cho, Shiwa-Gun, Yahaba, Iwate 028-3694 Japan; 2 Ultrasound General Imaging, GE HealthCare Japan, Hino-Shi, Japan

**Keywords:** Subharmonic-aided pressure estimation, Esophageal varices, Portal hypertension

## Abstract

**Background:**

Subharmonic-aided pressure estimation (SHAPE) is a technique for determining changes in ambient pressure. We aimed to analyze a novel SHAPE integrated into ultrasound diagnostic equipment to predict patients with liver cirrhosis at high risk of esophagogastric varices (EV).

**Methods:**

This prospective study included 111 patients with liver cirrhosis diagnosed between 2020 and 2023. We obtained liver stiffness measurements (LSM) and spleen stiffness measurements (SSM) using shear wave elastography and hepatic vein-portal vein (HV-PV) gradient using the SHAPE method. The EV risk was determined either as null, low, or high by esophagoscopy and Child–Pugh stage.

**Results:**

HV-PV gradient increased concordantly with the increase in EV risk (− 7.0 dB in null-risk, − 4.4 dB in low-risk, and − 2.0 dB in high-risk) with statistically significant difference among any two groups. The most appropriate cut-off value of the HV-PV gradient was − 3.5 dB, and sensitivity, specificity, and positive and negative predictive values were 80.0%, 89.0%, 80.0%, and 88.0%, respectively. The areas under the curve values for predicting the high-risk EV were 0.920, 0.843, and 0.824 for the HV-PV gradient, LSM, and SSM, respectively.

**Conclusions:**

The novel SHAPE system demonstrated high accuracy in identifying patients with liver cirrhosis at a high risk of EV.

**Supplementary Information:**

The online version contains supplementary material available at 10.1007/s00535-024-02161-4.

## Introduction

Portal hypertension is a cardinal complication of liver cirrhosis (LC) that causes severe outcomes, such as ascites, bleeding esophagogastric varices (EV), and hepatic encephalopathy [1–4]. The presence of clinically significant portal hypertension (CSPH) and variceal bleeding determines patient prognosis, influences mortality, and requires tailored diagnostic and therapeutic strategies [5–8]. The development and progression of EV exhibit considerable variability, with a prevalence of 30–40% in patients with compensated LC and 85% in those with decompensated LC. Variceal bleeding is associated with a substantial mortality rate of 20–40% [9–11]. While esophagogastroduodenoscopy (EGD) has been shown to be the most reliable procedure for the diagnosis of EV, it poses significant burdens regarding cost and patient discomfort and often necessitates sedation, suggesting a need for less invasive diagnostic alternatives [12], which can predict high-risk EV in patients with LC.

The Baveno VII Consensus Workshop criteria recommend that patients with a liver stiffness measurement (LSM) on vibration-controlled transient elastography (VCTE) of > 20 kPa or a platelet count of < 150 × 10^9^ /L should undergo endoscopy for screening of EV [8]. LSM and spleen stiffness measurement (SSM) using VCTE or shear wave elastography (SWE) have been described as cornerstone noninvasive tools for risk stratification and clinical decision-making in patients with CSPH to determine the risk of EV [13–17]. Furthermore, an alternative technique referred to as subharmonic-aided pressure estimation (SHAPE), which assesses harmonic signals from microbubbles to determine changes in ambient pressure, has been proposed [18–20]. Because SHAPE can detect variations in portal vein (PV) blood pressure and hepatic venous pressure gradient (HVPG), it has been suggested as a promising noninvasive modality for assessing portal hypertension [21–23]. However, limited evidence has confirmed the clinical applicability of SHAPE for the measurement of HVPG. In addition, current implementations of SHAPE prompt offline processing, a limitation that persists in general ultrasound (US) systems.

In this study, we added technical improvements to SHAPE to make it more suitable for clinical examination and integrated the revised version in a US diagnostic apparatus. We then prospectively examined whether the SHAPE method would provide greater precision for predicting high-risk varices in patients with LC.

## Methods

### Study participants

**Fig. 1:**
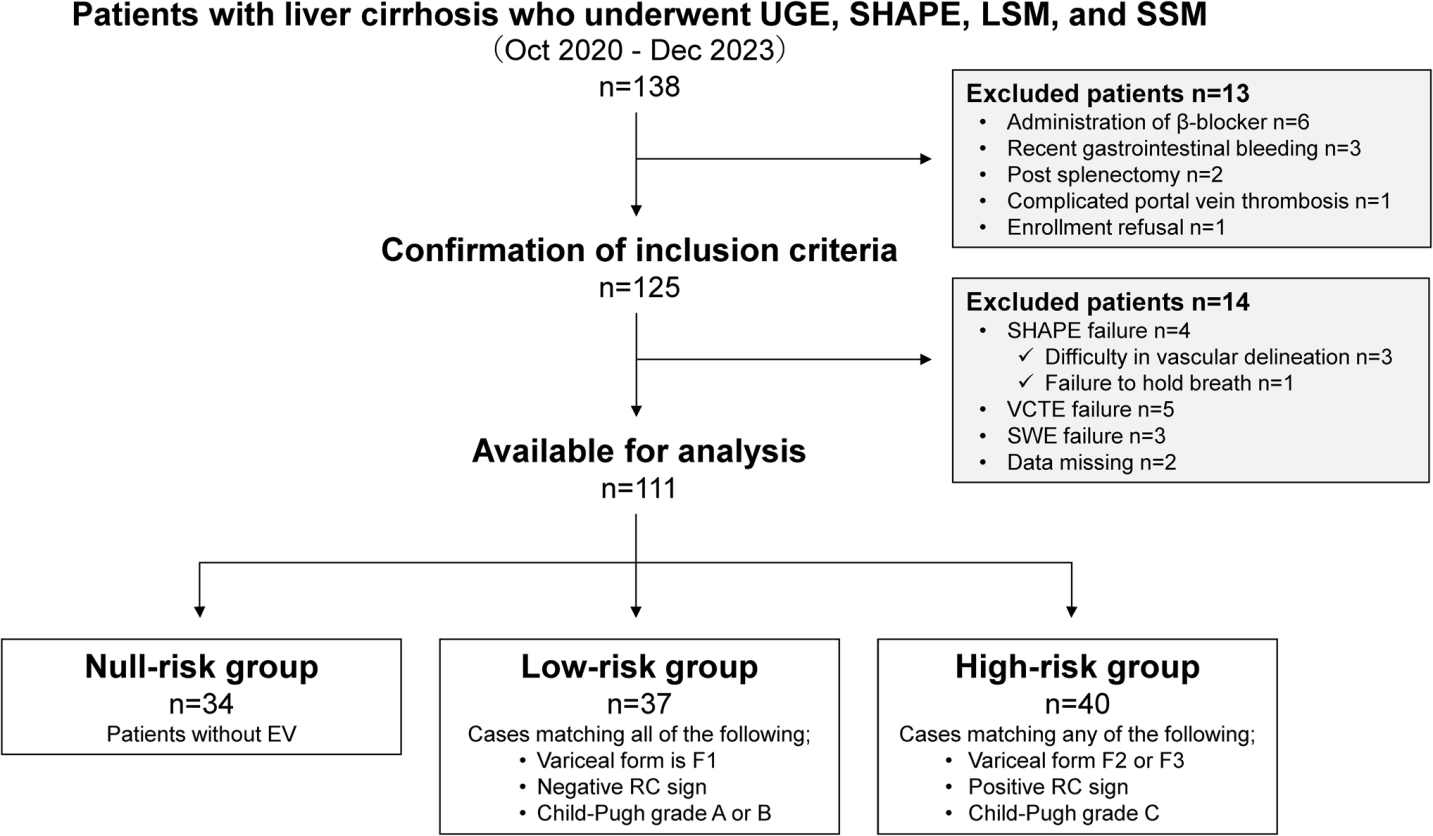
Flow chart of eligible patients with chronic liver disease. *EV* esophagogastric varices, *RC* red color, *SHAPE* subharmonic-aided pressure estimation, *SWE* shear wave elastography, *UGE* upper gastrointestinal endoscopy, *VCTE* vibration-controlled transient elastography

This single-center, prospective study was conducted between October 2020 and December 2023 (Fig. 1). The study population comprised consecutive patients with LC for whom EGD, SHAPE, LSM, SSM, and clinical data were available. The inclusion criteria were age ≥ 20 years, known LC, and willingness and ability to participate. LC diagnosis was made as described previously based on the results of histologic examination of the liver tissue or combined physical, laboratory, and radiologic findings [24, 25]. The etiologies of LC included viral hepatitis, alcohol-associated liver disease, and metabolic dysfunction-associated steatotic liver disease, with the diagnostic definitions detailed in Online Resource 1, Materials and Methods 1. Exclusion criteria were β-blocker use (*n* = 6), episodes of recent (< 3 months) gastrointestinal bleeding (*n* = 3), post-splenectomy (*n* = 2), PV thrombosis (*n* = 1) and refusal to enroll (*n* = 1).

The primary endpoint of this study was the predictive values of SHAPE, Baveno VII criteria, LSM, and SSM for the diagnosis of high-risk EV. All protocols of this study were approved by the Institutional Review Board of Iwate Medical University (approval number: MH2019-102). All patients provided written informed consent before the study according to the principles of the Declaration of Helsinki (revision of Fortaleza, 2013).

### SHAPE

SHAPE was performed using a LOGIQ E10 US system, with a C1-6-D probe (GE HealthCare, Wauwatosa, WI, USA) on the same day as LSM and SSM and before EGD. SHAPE analysis was performed by two hepatologists (H. K. and T. A. with at least 15 years of experience in abdominal US examinations), who were blinded to EGD findings and clinical data of the patients.

**Fig. 2:**
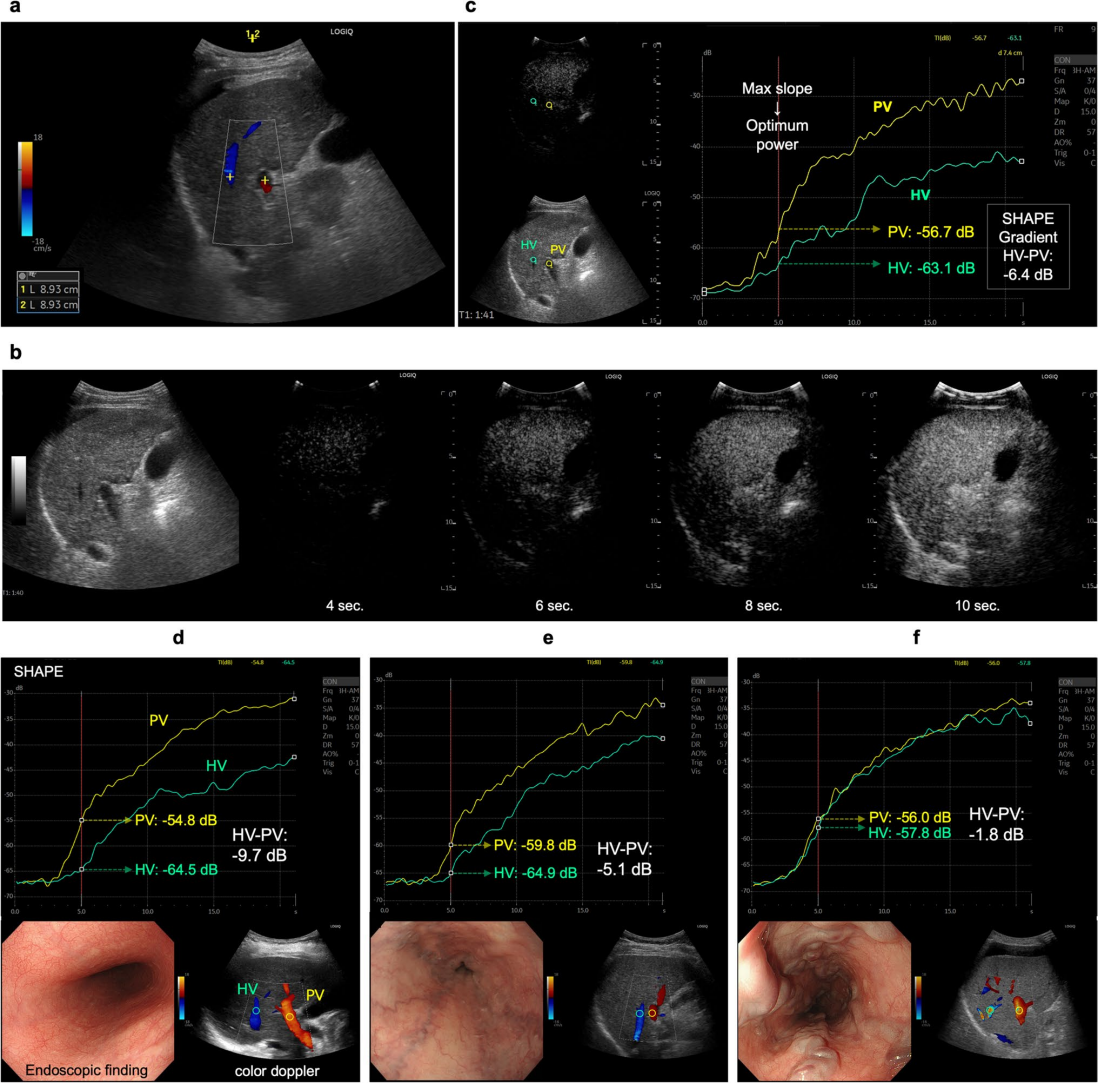
Identification of an ultrasound section that can simultaneously depict the right portal vein (PV) and hepatic vein (HV) at the same depth. **a** An image example of PV and HV identification using a color Doppler ultrasound examination. **b** Sample images of subharmonic contrast when the mechanical index is gradually increased. **c** An example of time intensity curve analysis, and the results for patients without varices (null-risk group); **d** those with straight, small-caliber varices (low-risk group); **e** and those with moderately enlarged varices (high-risk group) (**f**). The HV-PV values of the “Null-risk,” “Low-risk,” and “High-risk” groups are − 9.7, − 5.1, and − 1.8 dB, respectively

First, the US cross-sectional, right intercostal view with simultaneous right PV and right hepatic vein (HV) images was obtained (Fig. 2a). Second, cine recordings for SHAPE analysis were started 90 s after intravenous injection of Sonazoid^®^ (perfluorobutane microbubbles, GE HealthCare, Oslo, Norway) at a dose of 0.0075 mL/kg (Fig. 2b). To estimate the absolute hydrostatic pressure, the US sound pressure within the region of interest was kept constant. Since the efficacy of the acoustic power optimization method has been reported previously [20, 26], cine clips were recorded and stored for 20 s, during which the mechanical index (MI) incrementally increased by + 0.05 every 0.5 s under continuing breath-holding. The default parameters of the US system were, 57 dB for dynamic range, 37 cm for gain, and 15 cm for depth under full focus view. Third, time intensity curve analysis was performed using the software integrated into the US equipment. For the measurement, a region of interest with a 10-mm diameter for the PV and HV at the same depth was chosen. Subsequently, a graph depicting the relationship between the amplitudes and acoustic output of the subharmonic signal was automatically generated by the software. According to a previous report [27], the acoustic power corresponding to the subharmonic response most sensitive to changes in the hydrostatic pressure should be identified by its maximum slope. We selected the point of maximum slope of the PV as the optimum power (Fig. 2c). Finally, the HV minus the PV at the optimum power (HV-PV) was calculated for the SHAPE gradient measurement (Fig. 2d, e, f). The HV-PV was measured thrice for each patient to investigate the intra-observer variability. Furthermore, the HV-PV of all patients was successively evaluated by two investigators to determine interobserver variability.

### LSM and SSM

LSM was performed using a LOGIQ E10 with a C1-6-D probe and a FibroScan^®^ 502 Touch with an M or XL probe (Echosens, Paris, France). The LSM and SSM techniques and examination procedures have been previously described [28–32]. LSM and SSM are detailed in Online Resource 1, Materials and Methods 2 and 3.

### Evaluation of EVs

EV was evaluated endoscopically based on the published general rules for recording their endoscopic findings (second edition) [33]. The endoscopic findings were recorded in the form (F) of EV and the red color sign (RCS). EV was classified into four groups according to form and size. F0 lesions were lacking in varicose appearance; F1 lesions were straight, small-calibered varices; F2 lesions were moderately enlarged, beady varices; and F3 lesions were markedly enlarged, nodular, or tumor-shaped varices. RC signs of the EVs were graded 0, 1, 2, or 3 according to their density and distribution (RC0, absent; RC1, small in number and localized; RC2, intermediate between RC1 and RC3; RC3, large in number and circumferential). RC signs for gastric varices were graded as 0 or 1 (RC0, absent; RC, present with red-wale markings).

Based on the evaluation of varices and Child–Pugh grade, EV risk was stratified into three groups—no EV was regarded as null-risk; low-risk was defined as fulfilling the F1 form, negative RCS and Child–Pugh grade A or B; high-risk was defined as EV other than null and low risks.

### Statistical analyses

Based on the formula described by Karimollah et al. [34], we determined that the sample sizes for sensitivity and specificity were 95 and 110, respectively.

Data are presented as medians with interquartile ranges [25th and 75th percentiles]. Intra-observer agreement was assessed using the intraclass correlation coefficient (ICC) for the HV-PV gradient values. Thus, a sample size of 111 was finally selected. The Kruskal–Wallis test with Steel–Dwass post hoc tests was used to compare the three groups. The relationships between the clinical parameters and HV-PV values were examined using Spearman's rank correlation coefficients. The efficacy of the parameter in discriminating high-risk EV was evaluated using receiver operating characteristic curve analyses and category-free net reclassification improvement (cfNRI). Diagnostic performance variance across the models was explored by comparing the area under the curve (AUC) values analogous to the Harrell's concordance index (C-index). The highest Youden's J statistic was used to determine the optimal cutoffs for identifying the high-risk EV. Sensitivity, specificity, positive predictive values, negative predictive values, and positive and negative likelihood ratios were computed based on the AUC-derived cutoffs. AUC comparisons were performed using DeLong’s test. The model was internally validated using 1000 bootstrap samples. Calibration (agreement between observed and predicted outcomes) was assessed using calibration plots and a smoothing technique based on locally estimated scatterplot smoothing. Statistical significance was set at *P* < 0.05. All statistical analyses were conducted using EZR (version 1.53; Saitama Medical Center, Jichi Medical University), a graphical user interface for the R software (R Foundation for Statistical Computing).

## Results

### Participant characteristics

Of the 125 consecutive patients with LC, 12 failed their prospective US examinations. In the other two patients, there was loss of clinical data. Finally, 111 participants were enrolled in the present study (Fig. 1). There were seven participants with hepatocellular carcinoma. All tumors were less than 2 cm in diameter and had no vascular invasion. The participants were classified into “null- risk” (*n* = 28), “low-risk” (*n* = 37), and “high-risk” (*n* = 40) for EV. The participant demographics and clinical and laboratory features of the three groups are summarized in Table 1. Significant differences were found in aspartate aminotransferase and albumin values, prothrombin time, platelet count, FIB-4 index, Child–Pugh grade, model for end-stage liver disease score, endoscopic variceal form, and RC sign between the groups (*P* < 0.01).

**Table 1:** Clinical features and laboratory data of the study cohort

Characteristics	Null-risk group		Low-risk group		High-risk group	
No. of patients	34		37		40	
Sex (male, %)	20 (58.8)		25 (67.6)		23 (57.5)	
Age (years)	72.5 [62.5, 79.0]		71.9 [67.0, 78.0]		69.0 [62.8, 75.0]	
Etiology (HBV/HCV/ALD/MASLD/others)	5/12/9/7/1		3/15/11/8/0		3/5/17/12/3	
T.Bil (mg/dL)	0.9 [0.6, 1.1]		1.1 [0.5, 1.5]		1.2 [0.7, 1.9]	
AST (U/L)	29.5 [22.5, 40.1]		36.0 [28.2, 50.0]		44.0 [36.5, 53.0]*	
Alb (g/dL)	4.0 [3.5, 4.3]		3.7 [3.2, 4.1]*		3.2 [2.9, 3.7]*	
PT-INR	1.1 [1.0, 1.2]		1.2 [1.2, 1.3]		1.3 [1.2, 1.4]*	
Plt (× 10^4^/mm^3^)	11.1 [9.4, 14.2]		8.4 [6.9, 11.4]*		8.1 [5.9, 10.6]*	
FIB-4 index	4.5 [3.0, 5.6]		6.0 [4.4, 7.3]*		6.3 [4.5, 7.9]*	
Child–Pugh grade (A/B/C)	31/3/0		30/7/0		19/15/6*,**	
MELD score	8.0 [7.0, 9.0]		9.0 [8.0, 11.0]		10.0 [8.5, 13.0]*^,^**	
Endoscopic variceal form (F 1/2/3)	–		37/0/0		2/29/9**	
RC sign (RC 0/1/2/3)	–		37/0/0/0		1/26/10/3**	
Gastric varices, *n* (%)	–		6	(16.2)	7	(17.5)
HCC, *n* (%)	3	(8.8)	2	(5.4)	2	(5.0)

Values represent the median [25th, 75th percentile] or number (%)

*Alb* albumin, *ALD* alcohol-associated liver disease, *AST* aspartate aminotransferase, *EV* esophagogastric varices, *HBV* hepatitis B virus, *HCV* hepatitis C virus, *MASLD* metabolic dysfunction-associated steatotic liver disease, *PT* prothrombin, *T. Bil* total bilirubin

^*^P < 0.01 (compared with the null-risk group)

^**^ *P* < 0.01 (compared with the low-risk group)

^***^P < 0.05 (compared with the low-risk group)

### SHAPE, LSM, SSM, and percentages of agreement with the Baveno criteria by group

The US parameters and percentages of agreement with the Baveno criteria by group are presented in Table 2 and Online Resource 2, Supplementary Fig. 1. No statistically significant differences were observed in the HV across the three groups. In contrast, the PV in the low- and high-risk groups was significantly lower than that in the null-risk group (*P* < 0.01). The HV-PV increased in a stepwise manner from − 7.0 dB in the null-risk group to -4.4 dB in the low-risk group and up to − 2.0 dB in the high-risk group, each showing a statistically significant difference (*P* < 0.01). The percentages of agreement in the Baveno criteria were 8.8%, 18.9%, and 85.0% in the null-risk, low-risk, and high-risk groups, respectively (*P* < 0.01). LSMs determined by VCTE and SWE, and SSM were significantly higher in the high-risk group than in the other groups (*P* < 0.01). HV-PV was significantly correlated with the Child–Pugh grade, model for end-stage liver disease score, FIB-4 index, LSM (VCTE), LSM (SWE), SSM (SWE), variceal form (F value), and RC sign (*P* < 0.01) (Online Resource 3, Supplementary Table 1). The ICC for intra-observer agreement on HV-PV measurements was 0.801 (95% confidence interval [CI]: 0.697–0.870). Furthermore, the reproducibility of HV-PV among observers (for all patients) yielded an ICC of 0.796 (95% CI: 0.688–866).

**Table 2:** SHAPE, LSM, SSM, and the agreement with Baveno criteria by group

Characteristics	Null-risk group	Low-risk group	High-risk group
HV (dB)	− 50.8 [− 58.0, − 42.5]	− 52.0 [− 61.5, − 46.5]	− 53.0 [− 61.0, − 47.3]
PV (dB)	− 43.8 [− 50.0, − 35.3]	− 47.6 [− 55.2, − 41.7]*	− 51.1 [− 56.4, − 44.5]*
HV–PV (dB)	− 7.0 [− 9.0, − 5.6]	− 4.4 [− 5.5, − 3.6]*	− 2.0 [− 3.3, − 1.0]*^,^**
HV depth (cm)	8.1 [7.3, 9.4]	8.0 [7.5, 9.0]	7.6 [6.7, 8.7]
PV depth (cm)	8.1 [7.3, 9.4]	8.0 [7.4, 9.1]	7.6 [6.8, 8.7]
Baveno criteria met, *n* (%)	3 (8.8)	7 (18.9)	34 (85.0)*^,^**
LSM (VCTE) (kPa)	16.8 [12.9, 20.1]	18.3 [15.8, 22.2]	27.6 [21.4, 48.3]*^,^**
LSM (SWE) (kPa)	9.4 [7.6, 11.9]	10.0 [8.6, 13.1]	14.9 [12.7, 19.4]*^,^**
SSM (SWE) (kPa)	11.0 [8.9, 14.8]	11.6 [9.1, 18.6]	20.6 [16.4, 25.3]*^,^**

Values represent the median [25th and 75th percentiles] or number (%)

*HV* hepatic vein, *LSM* liver stiffness measurement, *SSM* splenic stiffness measurement, *SWE* shear wave elastography, *PV* portal vein, *VCTE* vibration-controlled transient elastography

^*^P < 0.01 (compared with the null-risk group)

^**^P < 0.01 (compared with the low-risk group)

^***^P < 0.05 (compared with the low-risk group)

### Correlation of HV-PV and other parameters with EV risk

**Fig. 3:**
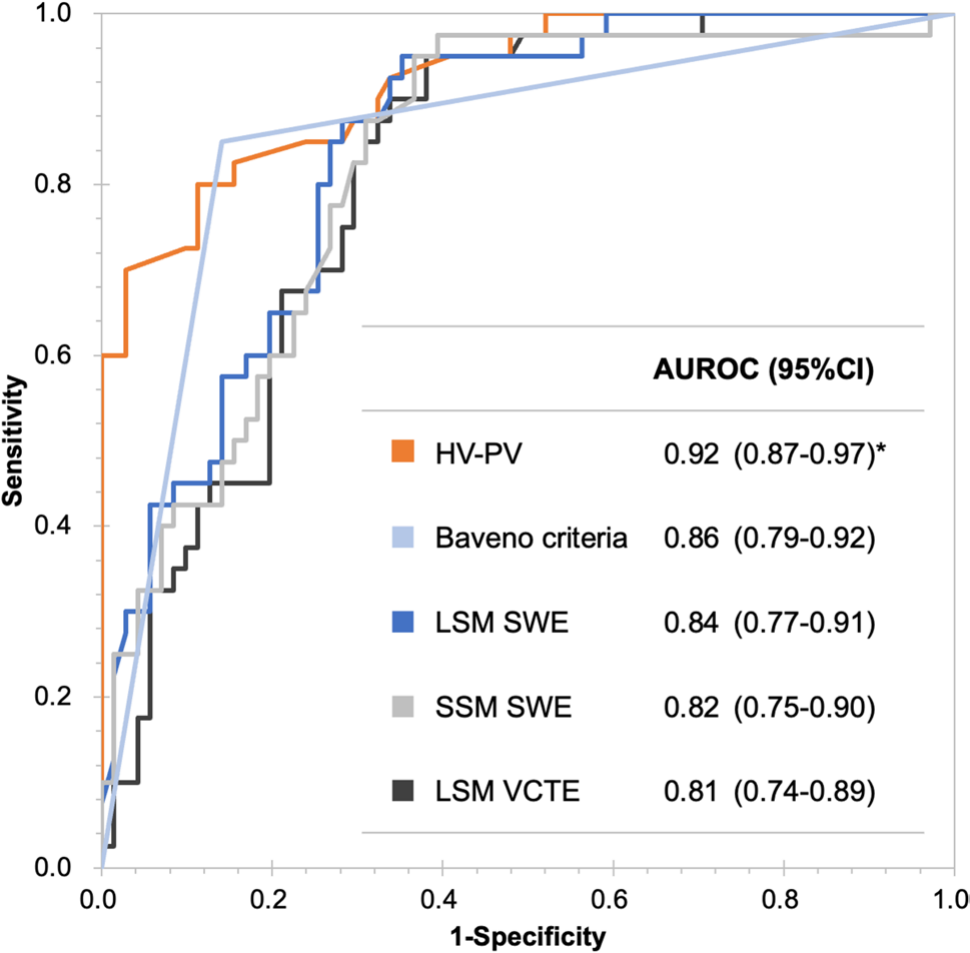
Receiver–operator curve (ROC) analysis for discriminating the high-risk group. The area under the ROC (AUROC) in discriminating the high-risk group was 0.92, 0.86, 0.84, 0.82, and 0.81 for HV-PV, Baveno criteria, LSM (SWE), SSM (SWE), and LSM (VCTE), respectively

AUC values for predicting the high-risk group were 0.920, 0.855, 0.813, 0.843, and 0.824 for HV-PV, Baveno criteria, LSM (VCTE), LSM (SWE), and SSM (SWE), respectively (Table 3, Fig. 3). Differences in the correlation between HV-PV and the Baveno criteria, and in the correlation between HV-PV and LSM under SWE, were not statistically significant (*P* = 0.115 and *P* = 0.071, respectively). In contrast, the differences in the correlation between HV-PV and LSM under VCTE, and in the correlation between HV-PV and SSM under SWE, were statistically significant (*P* = 0.019 and *P* = 0.035, respectively). The most appropriate cut-off value for HV-PV in discriminating the high-risk group from the other groups was − 3.5 dB. Sensitivity, specificity, positive predictive value, and negative predictive value of HV-PV for determining the high-risk EV were 80.0%, 88.7%, 80.0%, and 88.4%, respectively.

**Table 3:** Performance of various parameters for discrimination of the high-risk group

	HV-PV	Baveno criteria	LSM (VCTE)	LSM (SWE)	SSM (SWE)
AUC (95% CI)	0.92 (0.87–0.97)*	0.86 (0.79–0.92)	0.81 (0.74–0.89)	0.84 (0.77–0.91)	0.82 (0.75–0.90)
Cutoff value	− 3.5 (dB)	met	19.2 (kPa)	10.7 (kPa)	13.7 (kPa)
Sensitivity	0.80 (0.65–0.90)	0.77 (0.63–0.87)	0.95 (0.83–0.99)	0.95 (0.82–0.99)	0.93 (0.79–0.98)
Specificity	0.89 (0.79–0.94)	0.91 (0.81–0.96)	0.62 (0.50–0.72)	0.65 (0.53–0.75)	0.63 (0.52–0.74)
PPV	0.80 (0.68–0.92)	0.85 (0.74–0.96)	0.58 (0.47–0.70)	0.60 (0.48–0.72)	0.59 (0.47–0.71)
NPV	0.88 (0.81–0.96)	0.86 (0.78–0.94)	0.95 (0.88–1.00)	0.96 (0.90–1.00)	0.94 (0.87–1.00)
LR +	7.10 (3.63–13.88)	8.63 (3.96–18.83)	2.49 (1.84–3.39)	2.70 (1.95–3.73)	2.53 (1.84–3.47)
LR-	0.23 (0.12–0.42)	0.25 (0.14–0.43)	0.08 (0.02–0.32)	0.08 (0.02–0.30)	0.12 (0.04–0.36)

*AUC* area under the receiver operating characteristic curve, *CI* confidence interval, *HV* hepatic vein, *LR* + positive likelihood ratio, *LR* negative likelihood ratio, *LSM* liver stiffness measurement, *NPV* negative predictive value, *PPV* positive predictive value, *PV* portal vein, *SSM* spleen stiffness measurement, *SWE* shear wave elastography, *VCTE* vibration-controlled transient elastography

^*^P < 0.05 (compared with LSM (VCTE))

### Internal validation using bootstrapping

The C-indices adjusted by bootstrapping for the HV-PV, Baveno criteria, LSM under VCTE, LSM under SWE, and SSM were 0.921, 0.856, 0.811, 0.840, and 0.826, respectively (Table 4). HV-PV showed better discrimination performance in the high-risk group than LSM (VCTE) or SSM (SWE) (*P* = 0.015 and *P* = 0.039, respectively).

**Table 4:** Internal validation using bootstrapping method

Parameters	C-index	*p* value
HV-PV	0.921	Reference
Baveno criteria	0.856	0.121
LSM (VCTE)	0.811	0.015
LSM (SWE)	0.840	0.075
SSM (SWE)	0.826	0.039

*HV* hepatic vein, *PV* portal vein, *LSM* liver stiffness measurement, *SSM* spleen stiffness measurement, *TE* transient elastography, *SWE* shear wave elastography

### Comparative value for discrimination of the high-risk group and HV-PV calibration evaluation

The cfNRI values with HV-PV as a reference for the Baveno criteria, LSM under VCTE, LSM under SWE, and SSM were 0.539 (95% CI: 0.167–0.911), 1.121 (95% CI: 0.803–1.439), 0.865 (95% CI: 0.514–1.216), and 0.937 (95% CI: 0.596–1.277), respectively, showing a statistically significant difference (*P* < 0.01) (Table 5). HV-PV calibration plot analysis revealed the calibration slope and intercept to be 0.977 and 0.011, respectively (Online Resource 2, Supplementary Fig. 2).

**Table 5:** Comparison of parameters to discriminate high-risk groups by cfNRI (HV-PV vs. others)

Compared parameters		cfNRI	95%CI	*p* value
HV-PV	Baveno criteria	0.539	(0.167–0.911)	< 0.01
	LSM (TE)	1.121	(0.803–1.439)	< 0.01
	LSM (SWE)	0.865	(0.514–1.216)	< 0.01
	SSM (SWE)	0.937	(0.596–1.277)	< 0.01

*cfNRI*, category-free net reclassification improvement, *HV* hepatic vein, *PV* portal vein, *CI* confidence interval, *LSM* liver stiffness measurement, *SSM* spleen stiffness measurement

## Discussion

The use of noninvasive diagnostic techniques for identifying high-risk EV is inevitable to mitigate the load of EGD for both patients with LC and endoscopists. Despite advancements in the Baveno VII consensus for managing portal hypertension, there remains inadequate evidence regarding its applicability and effectiveness in certain geographical contexts, including Japan. In response to this clinical challenge, we attempted to use a refined SHAPE methodology that demonstrated a high level of diagnostic precision in detecting high-risk EV, as evidenced by an AUC of 0.92. Our findings seem to underscore the potential of SHAPE as a robust, noninvasive diagnostic tool for assessing portal hypertension and its complications.

Previous studies have highlighted the efficiency of SHAPE in predicting CSPH, with a strong correlation with HVPG [21–23]. The efficiency of SHAPE in portal hypertension has been previously underscored, with studies indicating a strong correlation between the SHAPE gradient and the HVPG, the gold standard in assessing portal hypertension severity. In their seminal work, Gupta et al. reported that the SHAPE gradient between the PV and HV exhibited a robust correlation with direct HVPG measurements, with a correlation coefficient of 0.68, highlighting the potential of SHAPE to mirror the hemodynamic alteration characteristics of portal hypertension [22]. In our investigation, HVPG measurements were performed in a cohort of 10 participants, reaffirming the positive correlation between the HV-PV gradient and HVPG, with a correlation coefficient of 0.879, as depicted in Online Resource 2, Supplementary Fig. 3. This correlation reinforces the diagnostic value of SHAPE for portal hypertension and its associated complications. A notable advancement in our study was the adaptation of the SHAPE technique for compatibility with conventional US systems, broadening its applicability in clinical settings. This technical enhancement facilitates the broader adoption of SHAPE and signifies a pivotal step toward its integration into routine clinical practice for assessing portal hypertension and high-risk EVs.

**Fig. 4:**
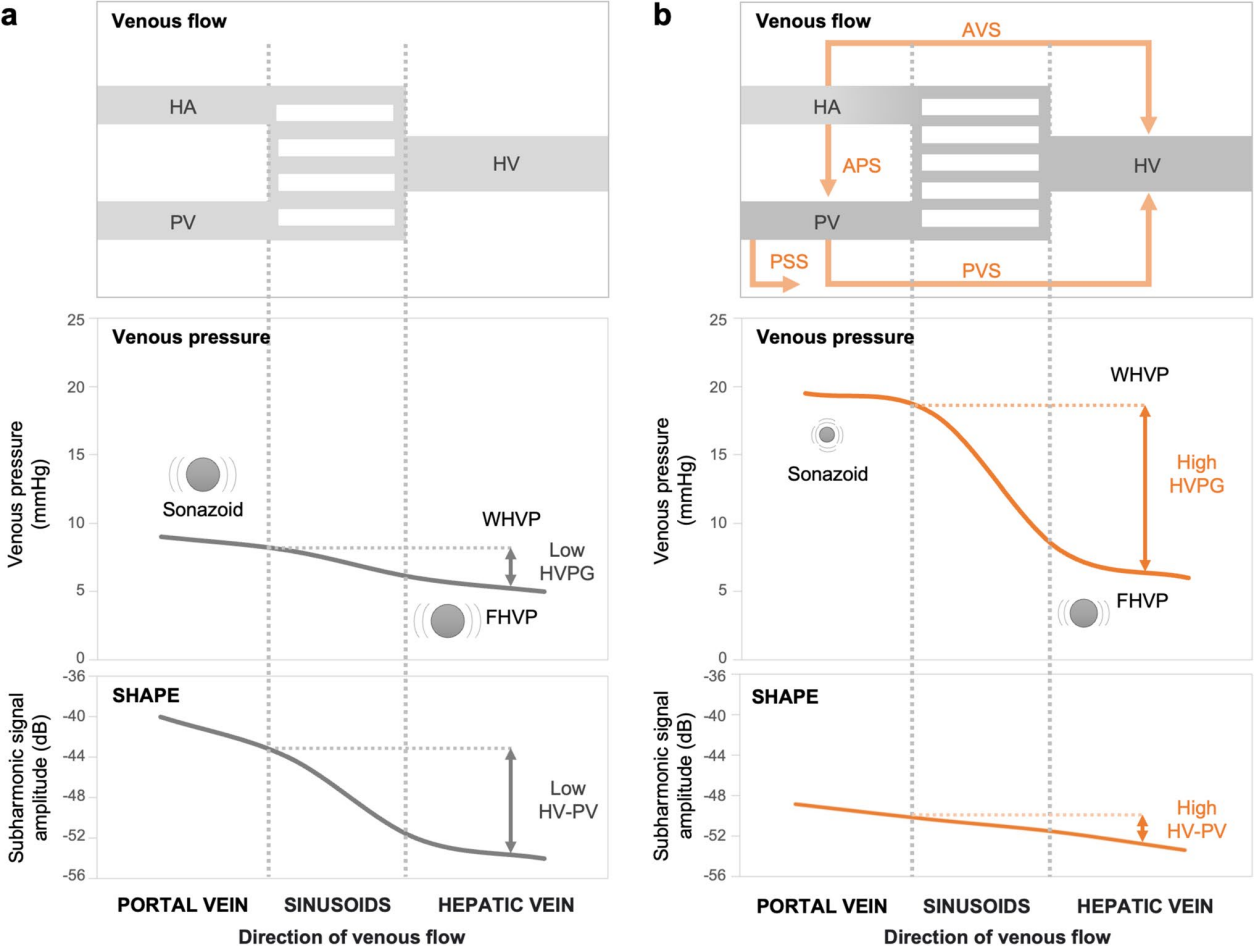
Schematic illustration of the hypothesized mechanisms of venous flow and pressure and the SHAPE gradient in clinically significant portal hypertension (CSPH). **a** Normal liver, and **b** CSPH. In CSPH, the PV subharmonic signal amplitude decreased owing to smaller microbubbles from increased portal pressure, and portosystemic shunting reduced the PV flow and lowered the microbubble count. HV signal reduction stems from extrahepatic collaterals, intrahepatic shunts, and HV pressure and flow changes owing to cirrhotic nodules, which affect microbubbles. The HV-PV gradient, which is essential in SHAPE, indicates the interaction between the PV and HV dynamics

We conclude that the increased HV-PV gradient may have been caused by the venous flow and pressure changes in portal hypertension with cirrhosis (Fig. 4). This study showed a significant decrease in the subharmonic signal amplitude of the PV flow with disease progression. It was assumed that this was primarily because of the reduced microbubble size owing to the increased portal pressure. Second, it was inferred that this was because the decreased PV flow owing to the portosystemic shunt with CSPH caused reduced microbubble counts. In patients with severe liver fibrosis and CSPH, a marked increase in vascular resistance prevents portal blood flow to the liver, resulting in the dilatation of anastomotic branches to the veins and formation of extrahepatic collateral circulation [35]. Additionally, the subharmonic signal amplitude of the HV tended to decrease with disease progression. This was assumed to be owing to extrahepatic collateral formation, increased intrahepatic shunts, and HV pressure and flow changes from the regenerative nodules of cirrhosis, which may affect the microbubble size and flow. We propose that HV-PV, a critical parameter of SHAPE, reflects the interplay between the PV and HV dynamics through the aforementioned mechanisms.

The factors influencing HV-PV measurements obtained via the SHAPE methodology remain inadequately defined. In this study, the AUC for predicting the high-risk group based on etiology was 0.931 (95% CI: 0.811–1.000) for the viral group (*n* = 43) and 0.913 (95% CI: 0.850–0.975) for the non-viral group (*n* = 68). There was no significant difference between the groups (*P* = 0.68), and the impact of etiology on SHAPE remains unclear. Additionally, in patients with decompensated cirrhosis, the HV-PV values may not always reflect the actual degree of CSPH, particularly in the presence of large umbilical veins or significant splenorenal shunts. Furthermore, arterioportal shunts can directly affect the HV-PV values by introducing arterial blood into the portal system, causing fluctuations in portal blood flow. Portal vein thrombosis, stenosis, or obstruction can also reduce portal blood flow, impacting HV-PV; thus, caution is advised when using SHAPE. Moreover, factors, such as multiple HV-HV shunts in the right hepatic vein, cardiovascular diseases (e.g., heart failure, pulmonary hypertension), patient physiological conditions (e.g., respiration, body position), and medications (e.g., beta-blockers, diuretics), can also directly influence the HV-PV values. Accumulating more cases and further clarifying the underlying pathophysiology are important future tasks.

This study had some limitations. First, the sample size was small, and large-scale prospective clinical studies are required to confirm the present findings. Second, compared with common dynamic computed tomography, contrast-enhanced ultrasonography (CEUS) is an operator-dependent examination. CEUS evaluates a single scanning plane at a time, which may not adequately represent the comprehensive situation of CSPH. CEUS and time intensity curve analysis applications are not available for all US instruments. Moreover, our study’s results obtained using the LOGIQ E10 US scanner with Sonazoid^®^ may not translate directly to those obtained with other US machines and other microbubble US contrast agents. The reproducibility and applicability of these results across different clinical settings may be worth considering in further studies. Third, SSM by VCTE was not included in the study protocol, and only SSM by SWE was performed in this study. In future, we plan to develop our research on SHAPE also incorporating SSM by VCTE comparisons. Finally, the influence of selection bias in our study cannot be ignored and may affect the interpretation of our findings.

In conclusion, our study offers compelling evidence supporting the use of SHAPE as a precise, noninvasive method for discriminating high-risk esophagogastric EV in patients with LC. SHAPE has potential as a noninvasive biomarker for esophagogastric EV, avoiding nonessential endoscopic evaluation. Further research is encouraged to build on these preliminary findings, expand the scope of the clinical utility of SHAPE, and confirm its effectiveness in broader patient populations.

## Supplementary Information

Below is the link to the electronic supplementary material.

Supplementary file1 (TIFF 13673 KB)

Supplementary file2 (TIFF 13673 KB)

Supplementary file3 (TIFF 13673 KB)

Supplementary file4 (DOCX 19 KB)

Supplementary file5 (DOCX 16 KB)

Supplementary file6 (DOCX 19 KB)
