# Correction: Novel subharmonic-aided pressure estimation for identifying high-risk esophagogastric varices

**DOI:** 10.1007/s00535-024-02194-9

**Published:** 2024-12-14

**Authors:** Hidekatsu Kuroda, Tamami Abe, Naohisa Kamiyama, Takuma Oguri, Asami Ito, Ippeki Nakaya, Takuya Watanabe, Hiroaki Abe, Kenji Yusa, Yudai Fujiwara, Hiroki Sato, Akiko Suzuki, Kei Endo, Yuichi Yoshida, Takayoshi Oikawa, Keisuke Kakisaka, Kei Sawara, Akio Miyasaka, Takayuki Matsumoto

**Affiliations:** 1 Division of Gastroenterology and Hepatology, Department of Internal Medicine, Iwate Medical University, Iwate Medical University School of Medicine, Nishitokuta 2-1-1, Yahaba-Cho, Shiwa-Gun, Yahaba, Iwate 028-3694 Japan; 2 Ultrasound General Imaging, GE HealthCare Japan, Hino-Shi, Japan

**Correction: Journal of Gastroenterology (2024)** 10.1007/s00535-024-02161-4

In the 'Funding' section of this article, the grant number was incorrectly given as 'JP20fk0210058' and should have been 'JP22fk0210113'.

The original article has been corrected.

