# Supplementary figures and images for: Novel subharmonic-aided pressure estimation for identifying high-risk esophagogastric varices
Source: J Gastroenterol. 2024 Oct 29;60(2):187–96. doi: 10.1007/s00535-024-02161-4 (PMC11794364; doi:10.1007/s00535-024-02161-4)

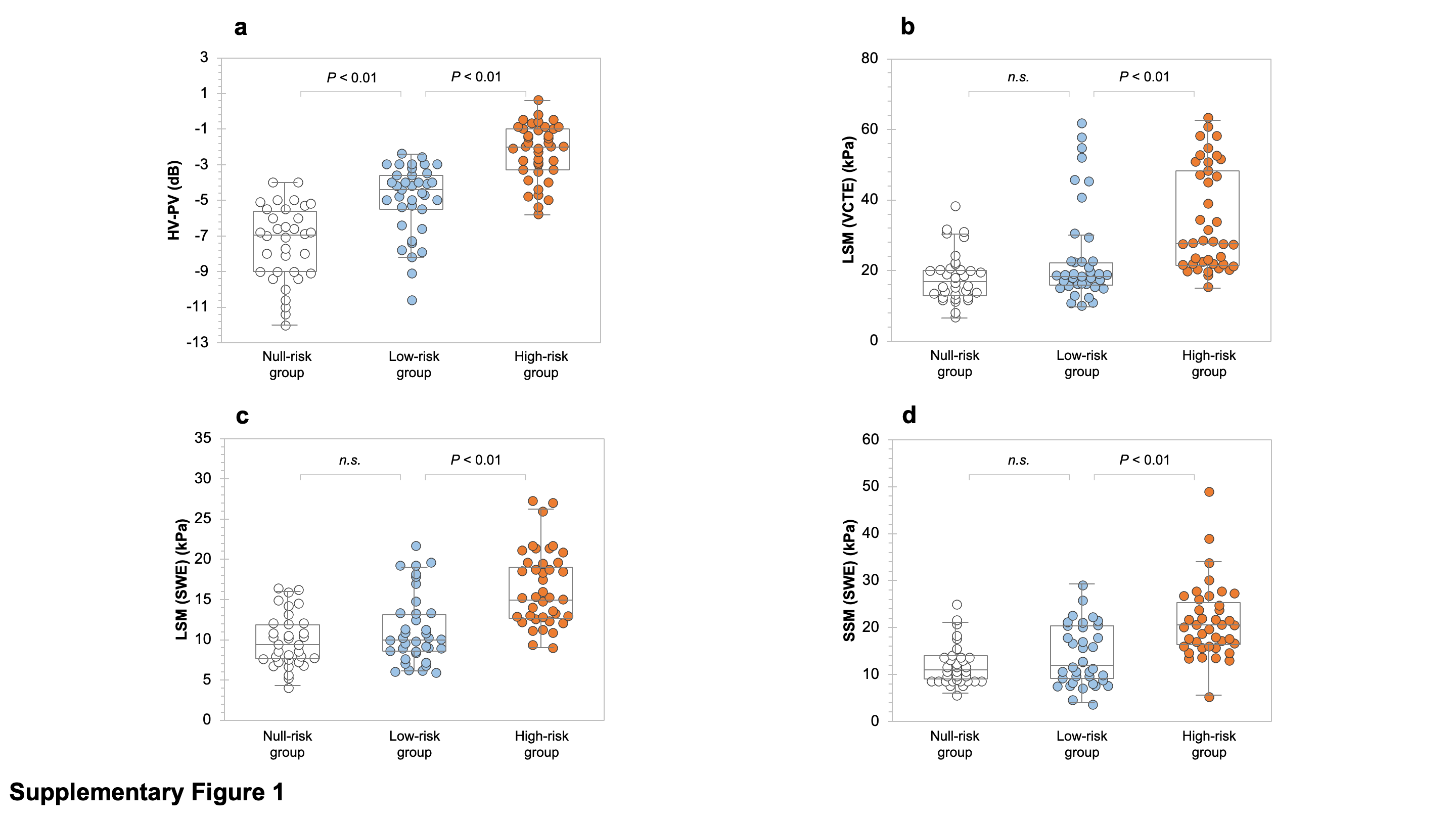

Supplement: Supplementary file 1 — Supplementary file1 (TIFF 13673 KB) [file 535_2024_2161_MOESM1_ESM.tiff]

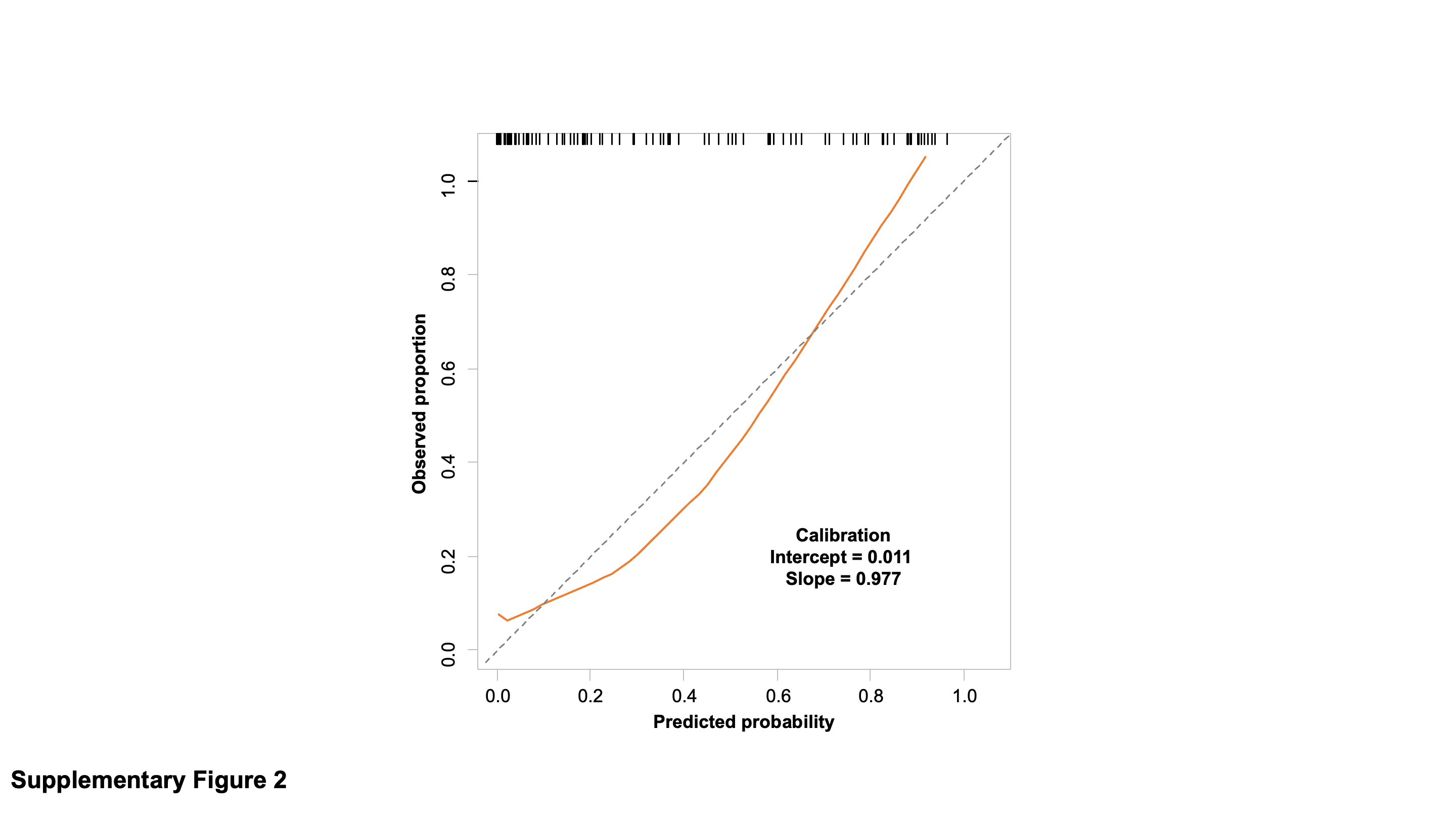

Supplement: Supplementary file 2 — Supplementary file2 (TIFF 13673 KB) [file 535_2024_2161_MOESM2_ESM.tiff]

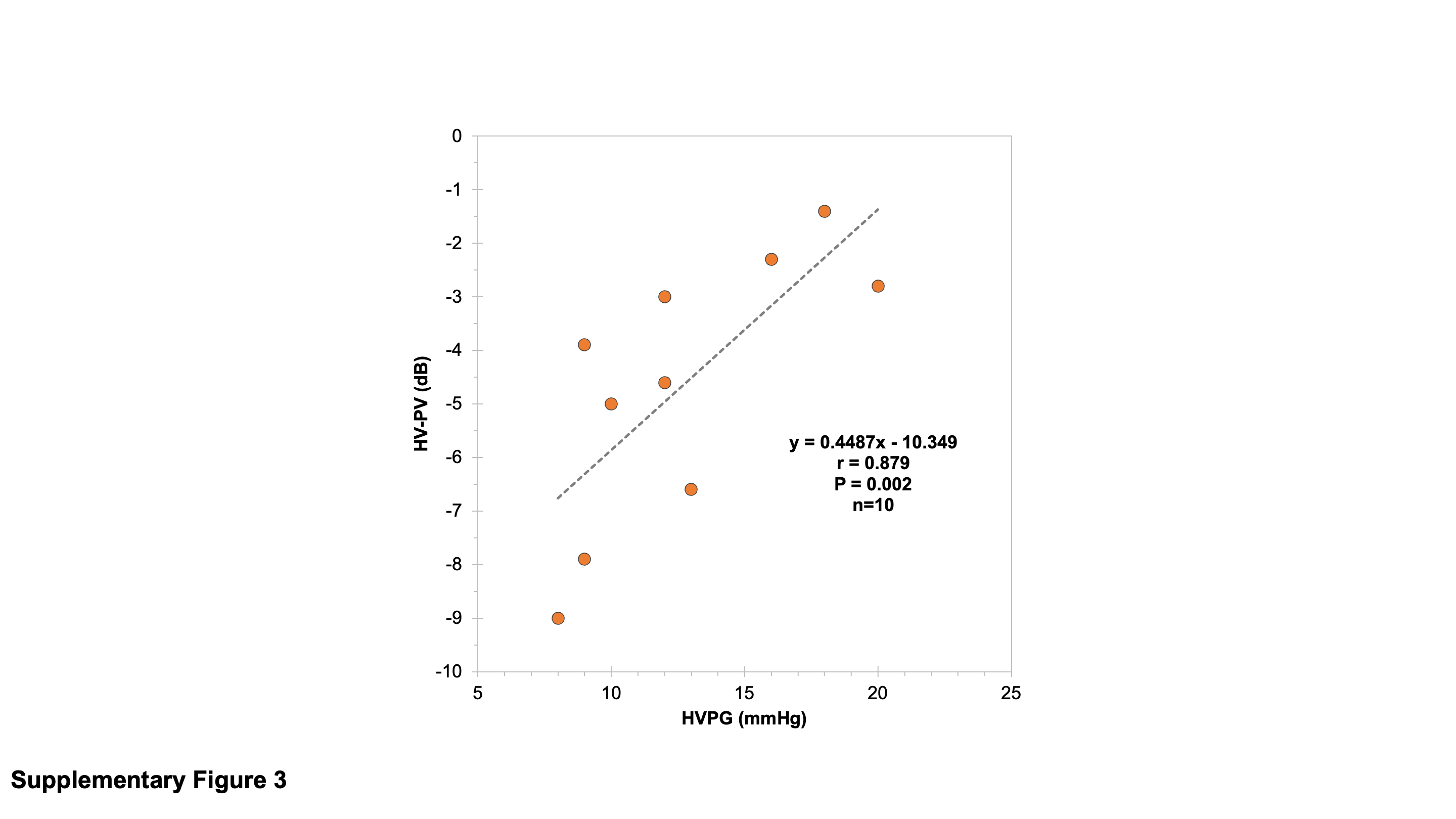

Supplement: Supplementary file 3 — Supplementary file3 (TIFF 13673 KB) [file 535_2024_2161_MOESM3_ESM.tiff]
