# Supplementary material for: Novel subharmonic-aided pressure estimation for identifying high-risk esophagogastric varices
Source: J Gastroenterol. 2024 Oct 29;60(2):187–96. doi: 10.1007/s00535-024-02161-4 (PMC11794364; doi:10.1007/s00535-024-02161-4)
Supplement: Supplementary file 4 — Supplementary file4 (DOCX 19 KB) [file 535_2024_2161_MOESM4_ESM.docx]

**Online Resource 1: Supplementary Material and Methods**

Novel Subharmonic-aided Pressure Estimation for Identifying High-risk Esophagogastric Varices

*Journal of Gastroenterology*

Hidekatsu Kuroda, Tamami Abe, Naohisa Kamiyama, Takuma Oguri, Asami Ito, Ippeki Nakaya, Takuya Watanabe, Hiroaki Abe, Kenji Yusa, Yudai Fujiwara, Hiroki Sato, Akiko Suzuki, Kei Endo, Yuichi Yoshida, Takayoshi Oikawa, Keisuke Kakisaka, Kei Sawara, Akio Miyasaka, Takayuki Matsumoto

**Corresponding Author**

Hidekatsu Kuroda, M.D., Ph.D., FRCP.

Division of Gastroenterology and Hepatology, Department of Internal Medicine, Iwate Medical University School of Medicine

E-mail: hikuro@iwate-med.ac.jp

**Materials and Methods 1: Diagnostic definitions for hepatitis C and B viral infections, alcohol-associated liver disease (ALD), and metabolic dysfunction-associated steatotic liver disease (MASLD)**

The study population comprised consecutive patients with liver cirrhosis (LC) who underwent upper gastrointestinal endoscopy, subharmonic-aided pressure estimation, liver stiffness measurement (LSM), and spleen stiffness measurement (SSM). LC was diagnosed based on the results of the histological examination of liver tissue or combined physical, laboratory, and radiological findings. The most common etiologies of LC are viral hepatitis, ALD, and MASLD. The diagnosis of hepatitis C and B viral infections was based on the detection of HCV RNA, hepatitis B surface antigen, and/or hepatitis B virus DNA. In this study, all cases of hepatitis B viral infection continued to be treated with nucleotide analogs (n=11, 100%). Additionally, all cases of hepatitis C viral infection achieved a sustained virological response after antiviral therapy (n=32, 100%). ALD was defined as a liver disease associated with alcohol intake of ≥30 and ≥20 g/day for men and women, respectively. Among the alcohol-related cases, 20 (54.1%) involved individuals who drank ≥60 g/day for men and ≥50 g/day for women. In contrast, 17 (45.9%) cases involved those who drank less than these amounts. MASLD was diagnosed in participants with hepatic steatosis who met at least one of the following cardiometabolic criteria: overweight (body mass index 23 kg/m^2^ or waist circumference >94 cm); impaired glucose tolerance (fasting blood glucose level ≥100 mg/dL, occasional blood glucose level ≥140 mg/dL, HbA1c value ≥5.7%, type 2 diabetes mellitus, or treatment with antidiabetic medicine); hypertension (blood pressure ≥130/85 mmHg); hypertriglyceridemia (triglyceride level ≥150 mg/dL); low high-density lipoprotein cholesterol (<40 mg/dL); or received any treatment related to these conditions.

**Materials and Methods 2: LSM**

LSMs were performed by two experienced hepatologists (H.K. and A.T., with 15 years of experience in abdominal ultrasound) blinded to the participants' upper endoscopy and clinical data. Measurements utilized a LOGIQ E10 with a C1-6-D probe (GE Healthcare, Wauwatosa, WI, USA) and a FibroScan^®^ 502 Touch with an M or XL probe (Echosens, Paris, France).

**LSM using shear wave elastography:** Participants who had fasted for 4 h were positioned supine with their right arm elevated. Liver targeting involved using real-time B-mode imaging through the intercostal spaces in the right liver lobe (Segment 5) to minimize pressure. A 30×15-mm color-coded map was placed at least 10 mm below the liver surface in a vessel-free zone. A circular region of interest (ROI) with a 10-mm diameter was used to display the mean liver stiffness within the sample box, avoiding vessels. The median of 10 LSMs, filtered by criteria including at least 10 valid measurements, a 60% success rate, and an interquartile range of <30% of the median LSM, represented liver stiffness. LSMs were considered invalid if elastic color signals covered less than 50% of the map.

**LSM using vibration-controlled transient elastography (VCTE):** VCTE was performed using a FibroScan 502 Touch. Subjects were assessed in the supine position with maximal right-arm abduction over the right hepatic lobe through the intercostal space. The initial measurements used the M probe, which was switched to the XL probe, as indicated by the automatic selection of the device—valid measurements required 10 measurements. Unreliability was defined as an IQR/median ratio of >30%, and technical failure was defined as fewer than 10 valid measurements.

**Materials and Methods 3: SSM**

SSM was performed by hepatologists H.K. and A.T. with 15 years of experience, using LOGIQ E10 (C1-6-D probe; GE Healthcare). Following a 4-h fast, the patients were placed in the supine position with the left upper extremity elevated for spleen access. Real-time B-mode imaging facilitated spleen visualization through the intercostal spaces. A 30×15-mm color-coded map, placed at least 10 mm beneath the spleen surface in a vessel-free zone, outlined a 10-mm diameter ROI for mean spleen stiffness, avoiding vessels. Spleen stiffness was based on the median of 10 valid SSMs, with invalid measurements if elasticity signals covered <50% of the map. Criteria for data inclusion were ≥10 valid measurements, a success rate of ≥60%, and an IQR of <30% of the median SSM.
