# Supplementary material for: Novel subharmonic-aided pressure estimation for identifying high-risk esophagogastric varices
Source: J Gastroenterol. 2024 Oct 29;60(2):187–96. doi: 10.1007/s00535-024-02161-4 (PMC11794364; doi:10.1007/s00535-024-02161-4)
Supplement: Supplementary file 5 — Supplementary file5 (DOCX 16 KB) [file 535_2024_2161_MOESM5_ESM.docx]

**Online Resource 2: Supplementary Figures**

Novel Subharmonic-aided Pressure Estimation for Identifying High-risk Esophagogastric Varices

*Journal of Gastroenterology*

Hidekatsu Kuroda, Tamami Abe, Naohisa Kamiyama, Takuma Oguri, Asami Ito, Ippeki Nakaya, Takuya Watanabe, Hiroaki Abe, Kenji Yusa, Yudai Fujiwara, Hiroki Sato, Akiko Suzuki, Kei Endo, Yuichi Yoshida, Takayoshi Oikawa, Keisuke Kakisaka, Kei Sawara, Akio Miyasaka, Takayuki Matsumoto

**Corresponding Author**

Hidekatsu Kuroda, M.D., Ph.D., FRCP.

Division of Gastroenterology and Hepatology, Department of Internal Medicine, Iwate Medical University School of Medicine

E-mail: hikuro@iwate-med.ac.jp

**Supplementary Fig. 1 Comparison of ultrasound parameters among the three groups**

The HV-PV increased in steps of -7.0 dB in the null-risk group, -4.4 dB in the low-risk group, and -2.0 dB in the high-risk group, each showing statistically significant differences (P<0.01). The LSM values using VCTE and SWE and SSE value using SWE were significantly higher in the high-risk group than in the other groups (P<0.01).

**Supplementary Fig. 2 Calibration plot results for HV-PV**

The calibration slope and intercept were 0.977 and -0.011, respectively.

**Supplementary Fig. 3 Correlation between HV-PV and HVPG**

The HV-PV gradient and HVPG, with a correlation coefficient of 0.879.
