# Supplementary material for: Novel subharmonic-aided pressure estimation for identifying high-risk esophagogastric varices
Source: J Gastroenterol. 2024 Oct 29;60(2):187–96. doi: 10.1007/s00535-024-02161-4 (PMC11794364; doi:10.1007/s00535-024-02161-4)
Supplement: Supplementary file 6 — Supplementary file6 (DOCX 19 KB) [file 535_2024_2161_MOESM6_ESM.docx]

**Online Resource 3: Supplementary Table 1**

Novel Subharmonic-aided Pressure Estimation for Identifying High-risk Esophagogastric Varices

*Journal of Gastroenterology*

Hidekatsu Kuroda, Tamami Abe, Naohisa Kamiyama, Takuma Oguri, Asami Ito, Ippeki Nakaya, Takuya Watanabe, Hiroaki Abe, Kenji Yusa, Yudai Fujiwara, Hiroki Sato, Akiko Suzuki, Kei Endo, Yuichi Yoshida, Takayoshi Oikawa, Keisuke Kakisaka, Kei Sawara, Akio Miyasaka, Takayuki Matsumoto

**Corresponding Author**

Hidekatsu Kuroda, M.D., Ph.D., FRCP.

Division of Gastroenterology and Hepatology, Department of Internal Medicine, Iwate Medical University School of Medicine

E-mail: hikuro@iwate-med.ac.jp

**Supplementary Table 1:** Correlation between HV-PV and clinical parameters

| **Parameters** | ***r*** | | **p-value** |
| --- | --- | --- | --- |
| **Sex** | 0.029 | 0.756 | |
| **Age** | -0.152 | 0.110 | |
| **Etiology** | 0.202 | 0.059 | |
| **T.Bil** | 0.254 | 0.007 | |
| **AST** | 0.373 | <0.001 | |
| **Alb** | -0.279 | 0.003 | |
| **PT-INR** | 0.166 | 0.081 | |
| **Plt** | -0.278 | 0.003 | |
| **Child-Pugh score** | 0.346 | <0.001 | |
| **MELD score** | 0.256 | 0.007 | |
| **FIB-4 index** | 0.309 | 0.001 | |
| **LSM (VCTE)** | 0.505 | <0.001 | |
| **LSM (SWE)** | 0.517 | <0.001 | |
| **SSM (SWE)** | 0.426 | <0.001 | |
| **Endoscopic variceal form** | 0.577 | <0.001 | |
| **RC sign** | 0.659 | <0.001 | |
| **Gastric varices** | 0.167 | 0.080 | |
| **HCC** | 0.040 | 0.671 | |

Abbreviations: Alb, albumin; AST, aspartate aminotransferase; FIB-4; Fibrosis-4; HCC, hepatocellular carcinoma; INR, international normalized ratio; LSM, liver stiffness measurement; MELD, Model for End-Stage Liver Disease; Plt, platelet; PT, prothrombin time; RC, red color; SSM, spleen stiffness measurement; T.Bil, total bilirubin; TE, transient elastography

n coefficient of 0.879.
